# Automatic aortic valve landmark localization in coronary CT angiography using colonial walk

**DOI:** 10.1371/journal.pone.0200317

**Published:** 2018-07-25

**Authors:** Walid Abdullah Al, Ho Yub Jung, Il Dong Yun, Yeonggul Jang, Hyung-Bok Park, Hyuk-Jae Chang

**Affiliations:** 1 Division of Computer and Electronic Systems Engineering, Hankuk University of Foreign Studies, Yongin, South Korea; 2 Department of Computer Engineering, Chosun University, Gwangju, South Korea; 3 Brain Korea 21 Project for Medical Science, Yonsei University, Seoul, South Korea; 4 Yonsei-Cedars Sinai Integrative Cardiovascular Imaging Research Center, Yonsei University Health System, Seoul, South Korea; 5 Division of Cardiology, Cardiovascular Center, Myongji Hospital, Seonam University College of Medicine, Goyang, South Korea; 6 Division of Cardiology, Department of Internal Medicine, Severance Cardiovascular Hospital, Yonsei University College of Medicine, Seoul, South Korea; 7 Cardiovascular Research Institute, Yonsei University College of Medicine, Seoul, South Korea; Harvard Medical School, UNITED STATES

## Abstract

The minimally invasive transcatheter aortic valve implantation (TAVI) is the most prevalent method to treat aortic valve stenosis. For pre-operative surgical planning, contrast-enhanced coronary CT angiography (CCTA) is used as the imaging technique to acquire 3-D measurements of the valve. Accurate localization of the eight aortic valve landmarks in CT images plays a vital role in the TAVI workflow because a small error risks blocking the coronary circulation. In order to examine the valve and mark the landmarks, physicians prefer a view parallel to the hinge plane, instead of using the conventional axial, coronal or sagittal view. However, customizing the view is a difficult and time-consuming task because of unclear aorta pose and different artifacts of CCTA. Therefore, automatic localization of landmarks can serve as a useful guide to the physicians customizing the viewpoint. In this paper, we present an automatic method to localize the aortic valve landmarks using colonial walk, a regression tree-based machine-learning algorithm. For efficient learning from the training set, we propose a two-phase optimized search space learning model in which a representative point inside the valvular area is first learned from the whole CT volume. All eight landmarks are then learned from a smaller area around that point. Experiment with preprocedural CCTA images of TAVI undergoing patients showed that our method is robust under high stenotic variation and notably efficient, as it requires only 12 milliseconds to localize all eight landmarks, as tested on a 3.60 GHz single-core CPU.

## Introduction

Aortic valve stenosis is a well-known valvular heart disease worldwide [1]. It has a comparable prevalence relative to other heart diseases, especially in the older population, affecting 2.7% of patients over 65 years old [2, 3]. The popular surgical procedure undertaken in order to treat severe stenosis is the open-heart surgical method of aortic valve replacement [4, 5]. During this surgery, a prosthetic valve is deployed, replacing the diseased valve. However, approximately 30% of patients are not able to endure the surgical trauma as a result of old-age [6]. An alternative to this method is minimally invasive transcatheter aortic valve implantation (TAVI). In this procedure, the deployment of the prosthetic valve is aided by a catheter that may be implanted through the femoral artery, the aortic arch or a cut near the heart apex [7]. Since it was first introduced in 2002, the adoption rate of this procedure in order to treat aortic valve disease has been increasing rapidly [8, 9]. It has significantly reduced the mortality rate of patients who are physically unable to undergo open-heart surgery. It has also shown a comparable result for patients who are able to stand the surgical stress [10].

**Fig 1:**
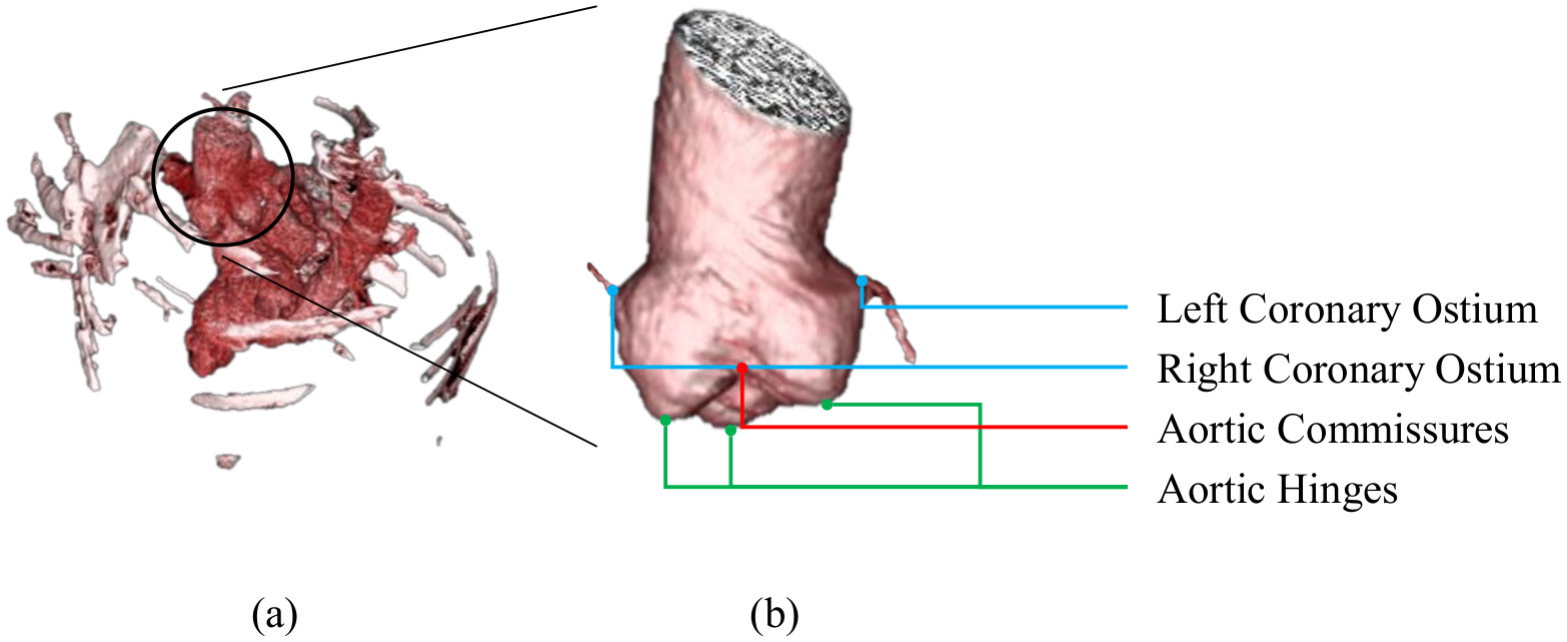
The aortic valve anatomy. (a) Rendered CT volume after thresholding to visualize the aortic valve. (b) An enlarged view of the aortic valve. The blue, green and red dots refer to the coronary ostia, the aortic hinges, and the aortic commissures, respectively. The commissure between the right-coronary and non-coronary hinges and the commissure between the left-coronary and non-coronary hinges are occluded in this view.

**Fig 2:**
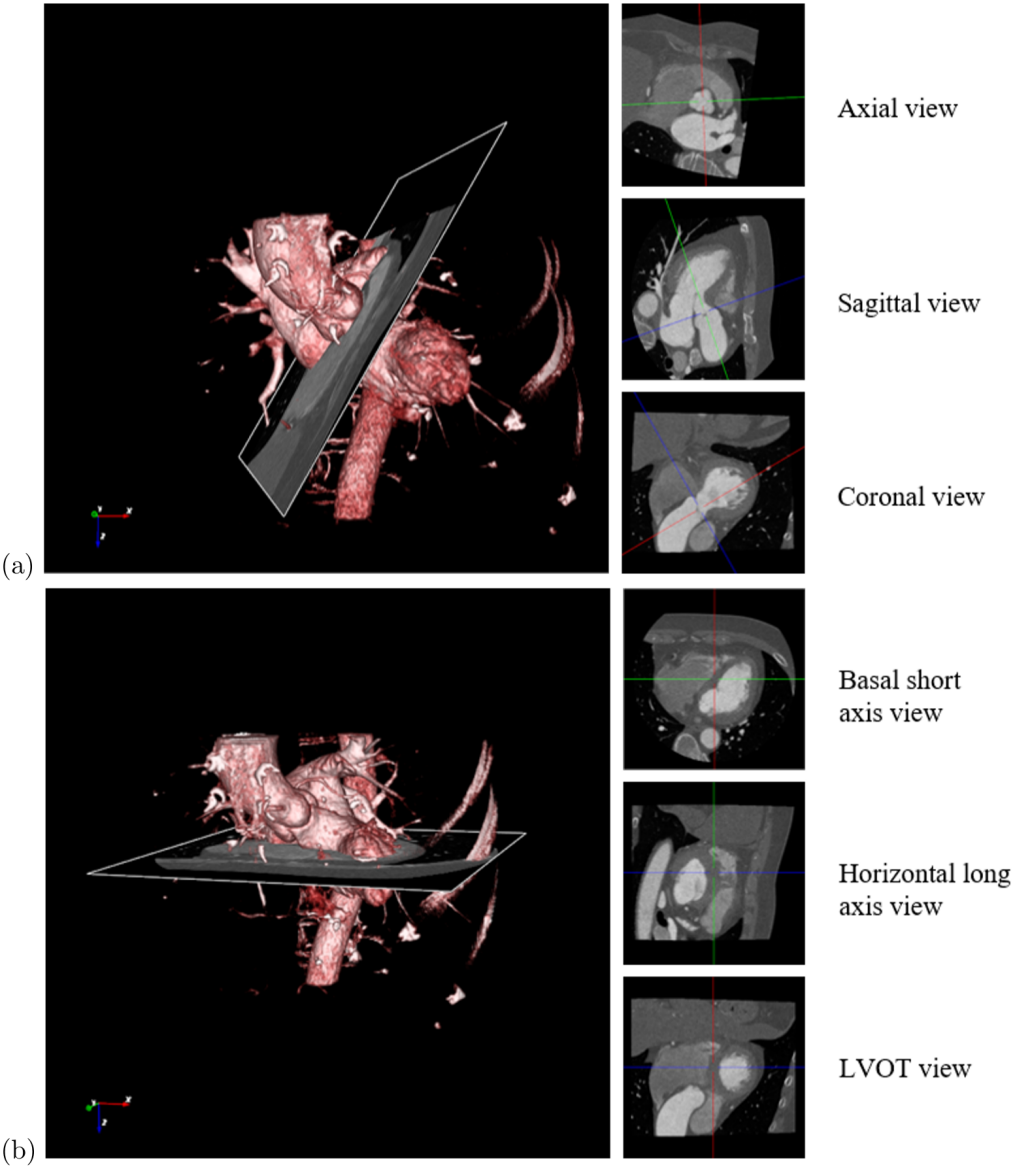
View customization for manually marking the landmarks and diagnosing. (a) Original view. (b) Customized view. Red, green and blue lines indicate the X, Y and Z axes, respectively. Transverse plane is carefully rotated about X an Y axes to have a view parallel to the aortic annulus because the aorta pose is not clear in the original view.

TAVI requires assessing a number of significant valve parameters before the surgical procedure. Fig 1 presents the aortic valve anatomy, where the valve landmarks are shown. Aortic valve annulus diameter is an important parameter for selecting a suitable prosthetic valve. The distance of the hinge plane to the coronary ostia is also a crucial parameter because an inaccurate measurement may block coronary circulation [11, 12]. Therefore, accurate localization of the eight aortic valve landmarks plays an important role. Contrast-enhanced coronary CT angiography (CCTA) is a frequently used imaging technique to obtain 3-D measurements for pre-operative preparation [13]. An automated method for localizing the landmarks in CT images can accelerate the measurement and standardize the planning procedure. Accurately marking the landmark position is a time-consuming process, which depends on the position and orientation of the aortic valve and the status of different artifacts in CCTA volumes. The conventional axial, coronal and sagittal views are not preferable because the orientation of the aortic valve is not clear. Usually, physicians prefer a view parallel to the aortic hinge plane to look through for measurement and diagnosis purpose. They customize the view by applying rotation about X and Y axes to make the transverse plane parallel to the hinge plane, as shown in Fig 2, where X, Y, and Z axes follow the right-to-left, posterior-to-anterior, and inferior-to-superior orientation. Therefore, an automated localization of the landmarks facilitates a quick customization of the view by automatically applying the rotation using the hinge locations, thus reducing time and effort of physicians.

With a limited work performed on aortic valve landmark localization, ostia localization required for analyzing the coronary is the major focus in prior research [14, 15]. Table 1 presents a summary of the existing works. Hennemuth et al. [14] propose to detect the ostium as the connecting point between the coronary artery and the segmented ascending aorta, where a tubular shape connected to the aorta is labeled as the artery. Nevertheless, connected component analysis exhibits instability in the presence of noise, producing a high failure rate. Coronary ostia can also be detected by tracking the coronary centerlines from the aorta surface [15]. However, the computationally expensive coronary tracing algorithm requires much more time because it has to operate for the entire aortic surface.

**Table 1:** Summary of the previous methods.

Author	Goal	Method	Data	Outcome	Computation time
Hennemuth et al. [14]	Aorta segmentation & ostia localization	Connected component analysis	61 CTA images	57% success	10-60 s on a 2 GHz Pentium M PC
Tek et al. [15]	Aorta Segmentation & ostia localization	Coronary centerline tracking	150 CTA images	98.7% accuracy	6 s on a 3.2 GHz PC
Ionasec et al. [16]	All the landmarks localization	Trajectory-based learning	65 TEE & 69 CT seqs.	1.45 mm error	4.8 s on a 3.2 GHz quad-core PC
Zheng et al. [17]	Aorta segmentation & landmarks localization	Marginal space learning	278 C- arm CT volumes	2.11 ± 1.34 mm error	0.3 s on a 2.3 GHz quad-core PC
Waechter et al. [18]	Aorta segmentation & ostia localization	Model-based adaptation	20 CT images	97.5% success	N/A
Gessat et al. [19]	Aorta segmentation and hinge & ostia localization	Manual marking	16 DynaCT images	N/A	N/A
Elattar et al. [20]	Aortic root segmentation and hinge & ostia localization	Intensity map	40 CTA volumes	2.81 ± 2.07 mm error	N/A

There has been less work on detecting landmarks other than ostia. A complete model of the valve is presented by Ionasec et al. [16], where all the landmarks are considered. The accuracy for detecting the landmarks in a static 3-D volume is not presented because their holistic approach is undertaken to detect landmarks trajectory in the entire temporal sequence. Previously, they proposed to detect each landmark individually in a static volume [21], which did not utilize the valve anatomy. A robust and efficient approach is proposed by Zheng et al. [17, 22], where a marginal space learning (MSL) is utilized to enable detecting a global object composed the landmarks, in terms of scale, orientation and position. From the detected global object, a rough estimation is made for each landmark, which is again refined using a local detector specific to each landmark. The optimal global object is found by a generalized Procrustes analysis (GPA)-based approach to have a minimized error in estimating landmark positions. However, according to [23], the convergence of means is not guaranteed in GPA. In addition, GPA is sensitive in order to detect and localize blunders in the dataset [24].

Some recent works on aorta segmentation for TAVI are extended in some ways to detect the landmarks as well. Waechter et al. [18] performed segmentation of the aorta and the cardiac chambers and proposed a comprehensive model of the aortic valve, as blended in the heart model. From the segmented aortic valve, the initial ostia-location is inferred, and the final detection is performed using model-based adaptation. Gessat et al. [19, 25] proposed a region growing based approach to segment the aorta. The user specifies the initial point, and manually marked the hinges and the ostia. Most recently, Elattar et al. [20] introduced an automatic method, where the aortic hinges and coronary ostia are detected on the surface of the aortic root. However, the surface of the root is obtained after segmentation based on thresholding followed by connected component analysis, which has robustness issue under image noise. Gao et al. [26] proposed a context-aware method to detect anatomical landmark required for initializing a deformable model using a two-layer regression model, where the first layer provides the initial displacements of the landmarks separately and the second layer refines them exploiting the context features provided by the former layer.

We propose an efficient automated method for localizing all the landmarks of the aortic valve in CT images. The CT images from different clinical sites, or sometimes from the same clinical sites, showed significant variation based on the scanning parameter settings. Therefore, conventional image processing techniques usually lack robustness under such variations. Machine learning techniques are a preferable choice to employ the deep knowledge implanted into the dataset annotated by an expert. Detection and localization problems were often treated as or relied on classification [27–29]. The classification techniques seem not to be an appropriate choice because the number of positive samples is negligible compared to the negative ones (e.g., for classifying the non-coronary hinge point in each volume, there is only one positive sample, and all of the other samples are negative). Consequently, the classifier tends to classify most of the test entries as being negative. A fair amount of work has been performed to handle sample bias or dataset imbalance in classification tasks. For example, [30] proposed a minimax estimation based model for learning a classifier that is able to adjust to sample selection biases. He et al. [31] provided a review of the existing technologies to solve the imbalanced data problem. Schapire et al. [32] introduced a method to recursively boost the accuracy of such weak learners. Zheng et al. [17, 22] estimated the position of the proposed global object by training a classifier using a probabilistic boosting tree, which they mention in their previous work [29]. They considered the voxels inside a 3 × 3 × 3 cube around the ground truth position to be the positive samples. However, the sample bias or imbalance is still dominant, which can cause bad performance or failure in boosting [33]. There are some recent works on localization, where it is no longer preferred to treat it as a classification. Criminisi et al. [34] introduced a regression forest-based method for efficient anatomy localization.

Instead of fitting the classification technique to our localization problem, we propose a colonial walk, which is a randomized regression tree-based machine-learning algorithm. Rather than learning voxel-wise binary class labels, we train a randomized regression tree to estimate voxel-wise unit directions to the target landmark. While testing, a colony of random walkers is initialized at different random voxels. Each walker in the colony then takes iterative steps exploiting the direction obtained from the trained tree, eventually moving around a target that can be obtained by taking the expectation of the step-positions. Each walker in the colony proposes its own target position. Thus, the colonial walk method can be viewed as a group of random tree walks initiated at different points. We propose to choose the walker with the minimum walk variance and take the expectation of its positions as the final target position. Random tree walk (RTW) was originally introduced in [35] for human body part localization from depth images and extended in [36]. However, a single random walk can result in higher error or inconsistency in landmark localization because high variation in CT images can misguide the walker. Sensitivity to the initial point can also affect the stability of the walk. The proposed colonial walk utilizes multiple walks initiated at multiple random points to improve the condition. The minimum walk variance i.e., the variance of the stepped positions of the walker decides the best-guided walk. Colonial walk has shown noteworthy improvement as observed in our comparative experiment with RTW. The proposed method showed a high localization accuracy in highly calcified preoperative CT volumes of TAVI patients. The proposed method also ensures high computational efficiency compared to previous landmark localization methods [17, 20, 22] because, in the voxel-wise classification framework, we need to test all of the voxels inside the considered volume, while the colonial walk only needs to traverse the tree for the voxels its walkers step into. Random walks are found to be implemented on labeling the pixels in multilabel, interactive image segmentation [37]. Spectral method was used to simulate random walk. However, it is a time-consuming process since the random walk problem has to be solved for each voxel.

Individual learning of eight regression trees corresponding to eight landmarks implies repeated use of the massive training set. Individual learning for each landmark also disregards the useful anatomical information of all of the landmarks belonging to the aortic valve. For efficient learning and maintenance of accuracy, we propose a two-phase optimized search space, the global estimation phase and the local estimation phase. Unlike the hierarchical approach of [17] (i.e., inferring position, scale and orientation of a global object), in our global estimation phase, we learn a globally representative point inside the valvular area from the whole CT volume. In the local estimation phase, we learn each landmark individually from a small area around the global point. For localizing the landmarks in the local phase, the global point serves as the initial point for the random walkers. Other image processing techniques (e.g., registration, segmentation etc.) can help detect the valve area in the global phase. However, the proposed colonial walk has a high computational efficiency taking milliseconds to perform the whole task.

We organize the rest of our paper as follows. Methods section describes our methodology in two subsections. In the first subsection, we describe the localization procedure of a point inside a 3-D CT volume using the proposed colonial walk. We describe the two-phase optimized search space learning model in the second subsection. In the Results section, we present our comparative experiment to evaluate our method. Finally, we mention our concluding remarks in the Discussion section.

## Methods

In the proposed method, we localize all eight landmarks of the aortic valve using a two-phase learning model, where a globally representative point inside an area that sufficiently surrounds the landmarks is first learned from the whole CT volume. Each landmark is then individually learned from a small area around that point. In each learning phase, we exploit a colonial walk for robust and efficient localization. In this section, we first explain the procedure of localizing a point in CT volume using the proposed colonial walk, and then we move to describing the two-phase learning scheme to localize all of the landmarks in detail.

### Localization of a point in a 3-D CT volume using colonial walk

Coronary CT angiography provides a 3-D image of the cardiac area. While previous methods relied on learning voxel-wise binary class labels in order to localize a point in CT images, colonial walk localizes a point using the knowledge of voxel-wise unit directions to the target point (ground truth). We train a randomized regression tree to learn the unit directions to the ground truth at each voxel. The trained tree stores the unit directions as clusters in the leaf nodes. Therefore, with a certain input at the root of the tree, we can reach a leaf node that meets the conditions of subsequent node parameters. Each leaf provides some representative unit directions with their corresponding probability. For estimating the position of a target point, colonial walk initializes a colony of walkers at different random points inside the input volume. We first describe the procedure for training the randomized regression tree, which is followed by the description of the colonial walk algorithm for localization in CT images.

#### Data preparation

Our input data are 3-D CT volumes. For a set of training volumes, *V* = {*V*_1_, *V*_2_, …, *V*_*n*_}, we have corresponding target ground truth points, ***P*** = {***p***_1_, ***p***_2_, …, ***p***_*n*_}. Here, *n* is the total number of CT volumes in the training set. We train our regression tree to learn voxel-wise unit directions. Therefore, the training samples are the voxels inside the CT volumes. Let us denote our training sample as follows:

$$\begin{array}{c} S=(v,x,\hat{u}) \end{array}$$ (1)

where *S* is a training sample, *v* represents volume index, ***x*** is the position vector of the sample in the corresponding volume, and $\hat{u}$ is the unit direction vector from the sample to the ground truth point of the corresponding volume, which is presented as follows:

$$\begin{array}{c} \hat{u}=\frac{p_{v}-x}{\left|\right|p_{v}-x\left|\right|} \end{array}$$ (2)

Here, ***p***_*v*_ is the position vector of the target in volume *V*_*v*_.

#### Feature

**Fig 3:**
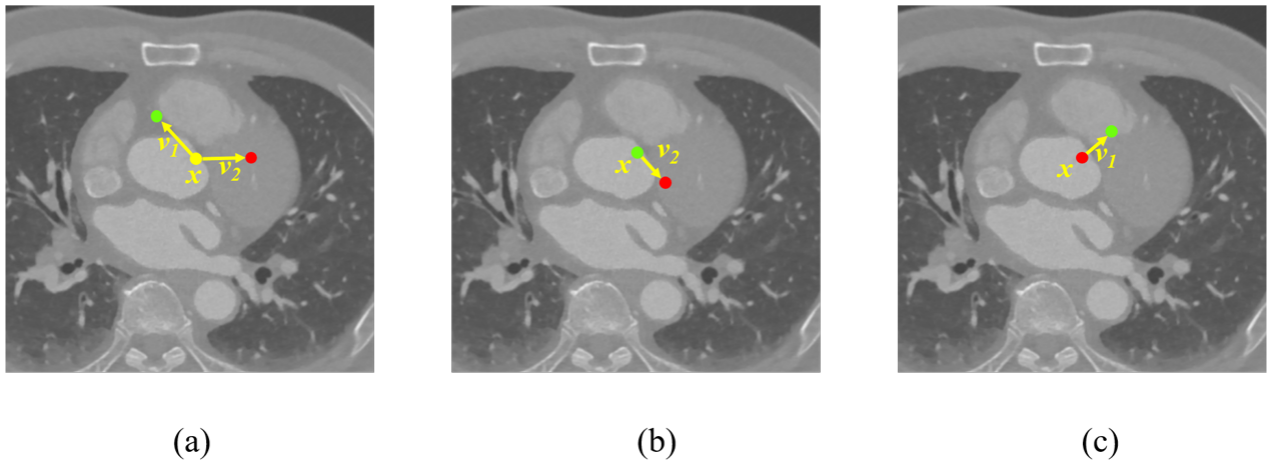
The voxel difference feature at *x*. The yellow dot refers to ***x*** i.e., the sample that needs to calculate the feature. The feature exploits the difference between the intensity at the green and red dotted positions. The distance vectors of these points from ***x*** are the feature offset parameters ***v***_**1**_ and ***v***_**2**_, respectively. In this figure, The component along Z-axis is considered zero for the parameters for visualization purposes. (a) The case of a random pair of the parameters. (b) and (c) The case of either one of the parameters being **0**.

We propose to use the simple voxel difference feature similar to the one used for human pose estimation in depth images [38]. This feature can be calculated efficiently and is able to provide significant and distinguishing information for the sample. The feature is calculated by taking the difference between two neighboring voxels of the sample. We can state the equation for feature calculation at ***x*** in volume *V*_*v*_ as follows:

$$\begin{array}{c} f_{\theta }\left(x\right)=\frac{I\left(x+v_{1}\right)-I\left(x+v_{2}\right)}{I(x)} \end{array}$$ (3)

where *I*(***x***) is the voxel intensity at ***x*** in volume *V*_*v*_ and *θ* = {***v***_**1**_, ***v***_**2**_} describes the offset ***v***_**1**_ and ***v***_**2**_, as shown in Fig 3.

The intensity difference value is normalized by the intensity of the current position and is therefore invariant to the amount of contrast agent. Zheng et al. [17] used Haar features to learn their classifier, which provides more information about the sample than the difference feature because the difference feature considers only two neighboring voxels to describe the sample. Though a single pair of neighbors is not sufficient, as the tree starts splitting, numerous unique pairs of offset are proposed, therefore providing a distinguishable measure for each sample. The simplicity of voxel difference feature is the advantage when learning regression tree, because we can try many different voxels in a limited time. By increasing the tree size, we get more complex combination of these simple features, and that makes regression tree robust even if the voxel difference feature is not be so robust. Difference feature has been proven effective in keypoint recognition [39].

#### Training

We perform training to create a binary tree by continuously splitting each node based on randomly generated features, thus minimizing the variation among the unit direction vectors $\hat{u}$ in the child nodes. A detailed review of randomized regression trees can be found in [40, 41].

At the parent node, *Q* is the set of input samples to be partitioned into two subsets (child nodes), *Q*_*l*_ and *Q*_*r*_. The objective function for the split is defined as follows:

$$\begin{array}{c} E_{Q}^{reg}\left(\phi \right)=\sum_{s\subset \{l,r\}}\sum_{\hat{u}\in Q_{s}}{\left|\right|\hat{u}-\bar{u_{s}}\left|\right|}^{2} \end{array}$$ (4)

$$\begin{array}{c} \bar{u_{s}}=\frac{1}{|Q_{s}|}\sum_{\hat{u}\in Q_{s}}\hat{u} \end{array}$$ (5)

Here, *ϕ* is the splitting parameter.

Our goal is to choose a parameter that minimizes this function at each split. We describe the overall training procedure as follows:

1. We randomly propose a set of split parameters, *ϕ* = (*θ*, *τ*), where *θ* = (***v***_1_, ***v***_2_) is the feature parameter (***v***_1_ and ***v***_2_ are the offset vectors), and *τ* is the threshold parameter.
2. We obtain the left and right subsets by partitioning the training examples, *Q*, for every instance of the proposed parameters, *ϕ*.
  $$\begin{array}{c} Q_{l}\left(\phi \right)=\left\{\left(v,x,\hat{u}\right)|f_{\theta }\left(x\right)<\tau \right\} \end{array}$$ (6)
  $$\begin{array}{c} Q_{r}\left(\phi \right)=Q\backslash Q_{l}\left(\phi \right) \end{array}$$ (7)
  where *Q*_*l*_(*ϕ*) and *Q*_*r*_(*ϕ*) are the left and right subsets, respectively. Here, *Q*_*l*_(*ϕ*)∪*Q*_*r*_(*ϕ*) = *Q* and *Q*_*l*_(*ϕ*)∩*Q*_*r*_(*ϕ*) = ∅. *f*_*θ*_(***x***) is calculated using Eq (3).
3. For every subset-pair obtained from the split parameters, we calculate our objective function, $E_{Q}^{reg}\left(\phi \right)$, using Eq (4).
4. Among the proposed split parameters, the parameter we choose to be the ultimate node split parameter is the one that minimizes the objective function.
  $$\begin{array}{c} \phi^{*}=\arg\min_{\phi }E_{Q}^{reg}\left(\phi \right) \end{array}$$ (8)
  where ϕ* is the ultimate split parameter for the current node.
5. We continue to split for *Q*_*l*_(ϕ*) and *Q*_*r*_(ϕ*) by following the above procedure until it can be classified as a leaf. We declare a node to be a leaf when the mean variance of the node become less than the *minimum variance* or the number of samples becomes less than the *minimum number of samples.*

Thus, in each leaf, we will have samples with directions to the ground truth. We use k-means clustering [42] to find the representative clustered directions at each leaf, i.e., the resultant centroids of the clusters. The desired value of k was 8 in our experiment and empty clusters created in the iterative process were dropped. The probability associated with each cluster is obtained from the assigned population.

#### Testing

**Fig 4:**
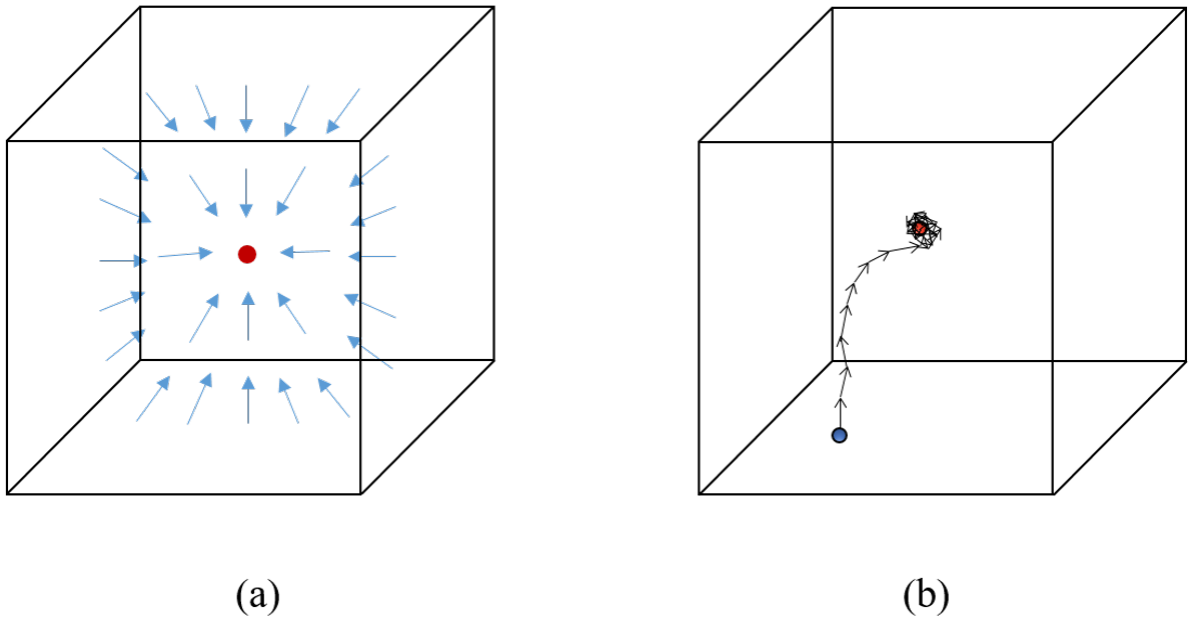
A single walk towards a target point in a 3D volume. The red dot is the target ground truth point. (a) The blue arrows refer to the learned unit directions to the ground truth at each voxel. (b) A walker starts from the blue point (i.e., the initial voxel) and updates to its next position taking a step towards the learned direction at the current position. After a certain steps, it starts moving around the ground truth point. The expectation of the step positions gives the target position.

Colonial walk exploits the trained regression tree to localize the target point of any given test volume. For a test volume, *V*_*test*_, colonial walk randomly chooses *N* random points, *X*_0_ = {***x***_1,0_, ***x***_2,0_, ………, ***x***_*N*,0_}, inside the volume, as the initial points of *N* random walkers of the colony. Each walker reaches a leaf of the trained regression tree starting from the root using (*V*_*test*_, ***x***_*i*,0_), by continuous branching according to the feature value for the corresponding node parameter. From the leaf, it randomly chooses a unit direction with its probability among the representative clustered directions. Then, the walker updates to a new point ***x***_*i*,1_ by taking a step of *dist*_*s*_ length in that direction. From that point, it again updates to a new point following the same procedure. After a certain number of iterations, random walks around the ground truth position can be noticeable as shown in Fig 4. Consequently, the walker makes a dense cloud marking its steps around the ground truth from a very close distance. Averaging all of the positions of the walker would give us that target ground truth point. *N* walkers in the colony makes proposition of *N* target positions, ${\bar{x}}_{:}$. The walker with the minimum walk variance, *σ*^2^(***x***_*ι*,:_) is chosen to be the successful walker in the colony. Therefore, the average position of the *ι*-th walker becomes the resultant target point. Algorithm 1 shows the overall testing procedure using the colonial walk.

**Algorithm 1:** The colonial walk

**Data:** Input CT Volume, *V*_*test*_,

initial pointset of the colony, *X*_0_ = {***x***_1,0_, ***x***_2,0_, ………, ***x***_*N*,0_},

regression tree, *T*_*reg*_, step length, *dist*_*s*_, and number of steps, *N*_*s*_,

**Result:** Target Point, ${\bar{x}}^{*}$

INITIALIZATION

$m=0,\bar{x_{:}}=0$;

**while** *m* < *N*_*s*_ **do**

 **for** *i*: 1 to *N* **do**

  Find leaf node *ζ* of *T*_*reg*_ using (*V*_*test*_, ***x***_*i*,*m*_).;

$\zeta =\{\left(p_{1},{\hat{u}}_{1}\right),\left(p_{2},{\hat{u}}_{2}\right)...,\left(p_{k},{\hat{u}}_{k}\right)\}$;

  Randomly choose ${\hat{u}}_{j}$ with probability *p*_*j*_;

  Update position using ${\hat{u}}_{j}$;

$x_{i,m+1}=x_{i,m}+{\hat{u}}_{j}.dist_{s}$;

$\bar{x_{:}}=\bar{x_{:}}+\frac{1}{N_{s}}x_{i,m+1}$;

  *m* = *m* + 1;

*ι* = arg min_*i*_ *σ*^2^(***x***_*i*,:_);

${\bar{x}}^{*}=\bar{x_{\iota }}$;

The walk variance, *σ*^2^(***x***_*i*,:_), refers to the variance of the stepped positions of the *i*-th walker, i.e., ***x***_*i*,*m*_, where 0 ≤ *m* < *N*_*s*_ and $x_{i,m}\epsilon N^{3}$. Consequently, $\sigma^{2}\left(x_{i,:}\right)\epsilon R^{3}$ has 3 components along X, Y and Z axes. We consider the magnitude of the variance and define the walk variance as follows:

$$\begin{array}{c} \sigma^{2}\left(x_{i,:}\right)=\sigma^{2}\left(x_{i,:}^{\alpha }\right)+\sigma^{2}\left(x_{i,:}^{\beta }\right)+\sigma^{2}\left(x_{i,:}^{\gamma }\right) \end{array}$$ (9)

where $x_{i,:}^{\alpha }$, $x_{i,:}^{\beta }$ and $x_{i,:}^{\gamma }$ refers to the X, Y and Z components of the step positions of the *i*-th walker. We take iterative approach to update the variance of each walker after each step.

In the original RTW method, a single tree walk decides the localization result. High variation lies in CT images because of different artifacts (e.g., staircase, motion, blooming etc.), patient and observer specific test parameters etc. There is a significant probability of presence of unknown voxels in the test volume from the learned regression tree point-of-view, which can misguide the walker resulting in an inaccurate localization result. This can happen because of an unknown initial voxel as well as an intermediary voxel on the walk. The colonial walk method exploits multiple random walks initiated at multiple random voxels to find the best-guided way to the ground truth position.

**Fig 5:**
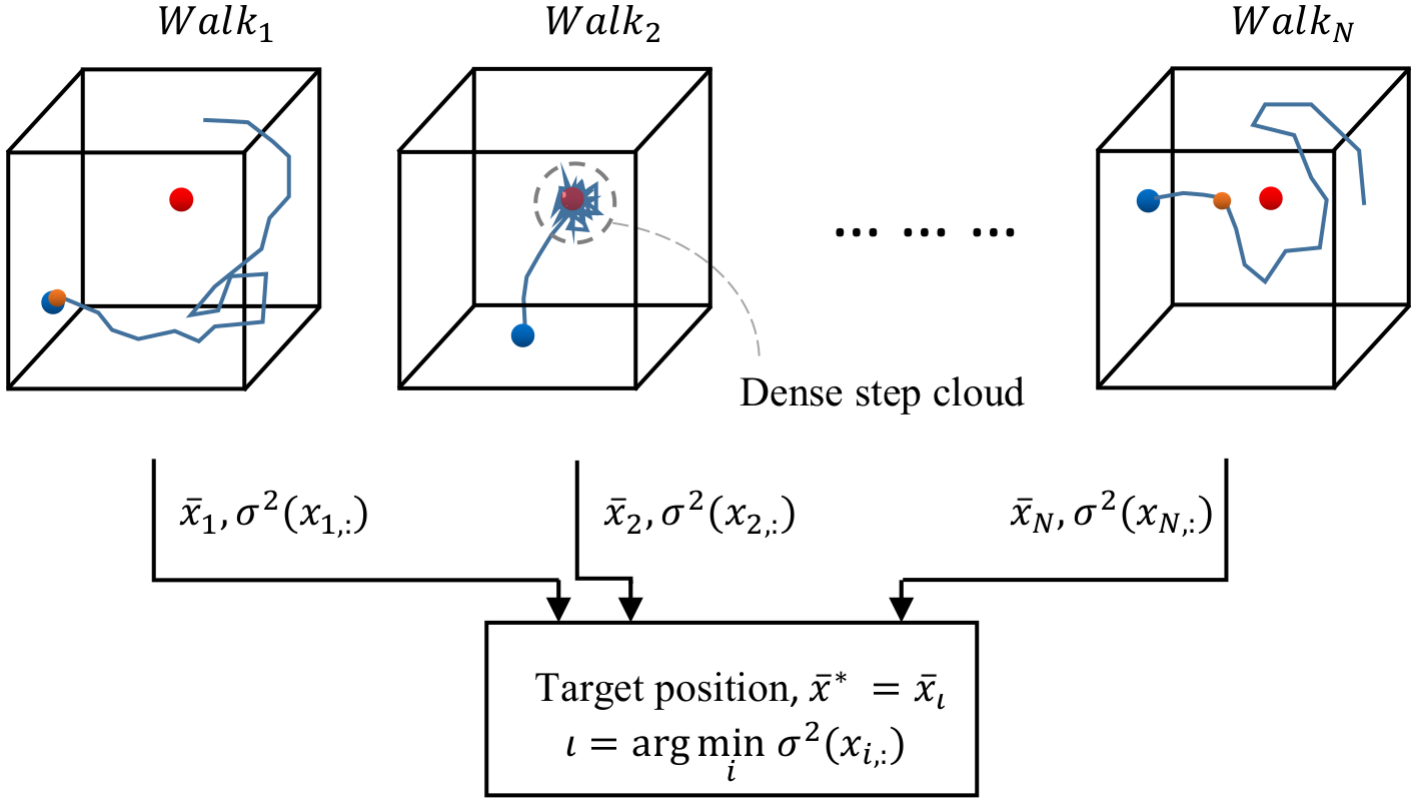
A colonial walk from multiple random points. The red dot is the target ground truth position. The orange dot refers to the first unknown point that misguides the walker. Initial point of the 1^st^ walk is unknown to the regression tree. The 2^nd^ walker converges and make dense step cloud around the target. The first misguider point of the N-th walk is not the initial point but a point through its way. The walker with the minimum walk variance is considered to be the best-guided walker.

The above defined walk variance is the key element that quantifies the guidance level of the walk. Usually, a walk shows high localization error when it fails to converge (i.e., fails to make any dense cloud around any point). Therefore, such walks show high walk variance. A well guided walk reach the dense cloud condition, moving around the ground truth position keeping a very small distance in between, showing a low walk variance consequently. Fig 5 shows the colonial walk method showing three major cases of walk.

### The two-phase learning model for localizing the aortic valve landmarks

Our ultimate objective is to localize eight landmarks of the aortic valve in CT images. We can learn any target point in a 3-D CT image using the colonial walk algorithm described in the previous subsection. The CT images we used in our experiment were of 288 slices on average, with each slice having 512 x 512 voxels (samples). The colonial walk needs to learn the unit directions to the target point (ground truth position), which is specific to each sample. However, it is computationally expensive to learn the directions for each sample from this vast search space. Sparse sampling can provide a solution to the issue by reducing the search space. Sparse sampling can also cause information loss and increase the localization error because the walkers might not be able to find enough guidance regarding the precise direction to reach the ground truth position accurately. Some approaches can be found in the literature to solve such scalability issue [43–45]. However, those approaches may be applicable to non-parametric machine learning algorithms.

Moreover, we have eight target points corresponding to eight landmarks of the aortic valve. The computation time even increases if we learn to localize these eight landmarks individually because we need to employ the whole search space repeatedly for estimating each landmark position. Individual detection also ignores the useful anatomical information of all of the landmarks being parts of the aortic valve. All eight landmarks are located close to each other, belonging to a relatively small area (i.e., the valve area). Prior detection of the valve area can reduce the computational cost and contribute to efficient individual learning of the landmarks because the colonial walk only requires utilizing the samples from the significantly reduced search space (i.e., the detected valve area) for all landmarks.

**Fig 6:**
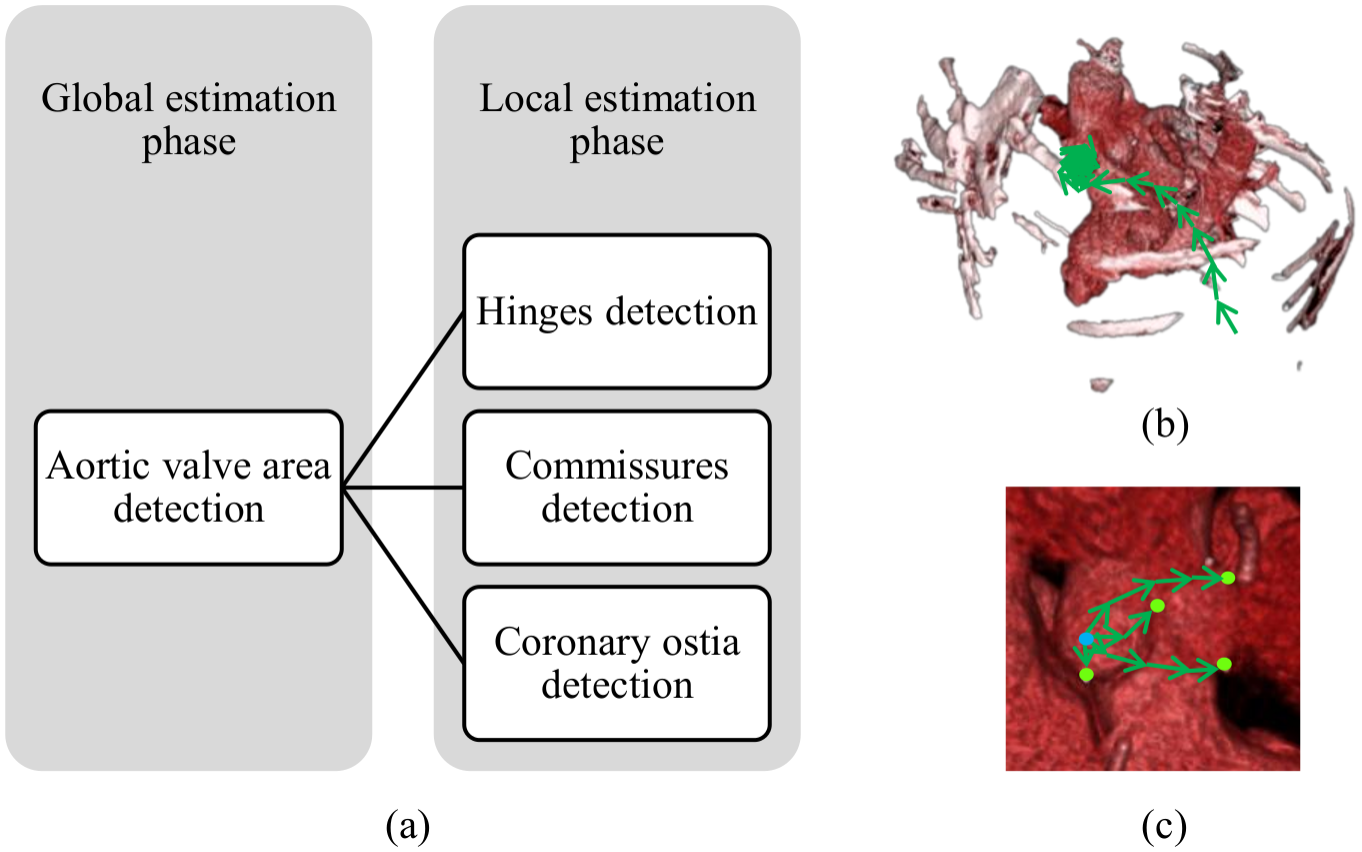
The proposed two-phase model for learning the aortic valve landmarks. (a) A representative point inside the valve area is detected in the global phase. In local phase, all the eight landmarks are localized from the estimated point in the global phase. (b) The point inside valve area is detected by colonial walk in the whole volume. (c) All the landmarks are localized locally by colonial walk from the globally estimated point.

We introduce a two-phase learning scheme for automatically localizing the landmarks of the aortic valve, considering the issues mentioned above. To improve robustness and efficiency in learning, we consider an area surrounding all of the landmarks of aortic valve. The first phase of our learning approach is the global estimation phase, where we learn to detect the considered landmark-surrounding area in the full search space. The second phase is the local estimation phase, where we learn to detect each landmark locally from the detected area. The proposed approach is further described in the following paragraphs in this section. Fig 6 shows the overall learning approach to localize all of the hinge points, commissure points, and coronary ostia.

#### Global estimation phase

This phase is the first phase of the proposed learning approach. As mentioned in the previous paragraph, we consider an area that sufficiently bounds all of the landmarks inside it. The second phase of estimating individual landmarks depends on the success of this phase.

Let us denote the set of landmarks as follows:

$$\begin{array}{c} X_{L}=\left\{h^{r},h^{n},h^{l},c^{\text{rn}},c^{\text{nl}},c^{\text{lr}},o^{r},o^{l}\right\} \end{array}$$ (10)

where ***h***^***r***^, ***h***^***n***^ and ***h***^***l***^ are the right-coronary, non-coronary and left-coronary hinge points, respectively. ***c***^***rn***^ is the commissure point between the right-coronary and non-coronary hinges. ***c***^***nl***^ is the commissure point between the non-coronary and left coronary hinges. ***c***^***lr***^ is the commissure point between the left-coronary and right-coronary hinges. ***o***^***r***^ and ***o***^***l***^ represent the right and left coronary ostium.

The colonial walk can find its path to the target if it is aware of the point of initialization. We do not need to detect the whole bounding area but only a point inside the area, which can serve as a useful point of initialization for the localization of all of the landmarks in the second phase. A cuboid of 150 × 150 × 60 voxels can sufficiently bound all of the landmarks in CT images used in our experiment. We fix any point inside that area as the target point, which seems roughly close enough to all of the landmarks and, more importantly, is easily distinguishable. This point can also be one of the landmarks (e.g., the non-coronary hinge point, ***h***^***n***^ because $-50<(x_{i}-h_{i}^{n})<50$ voxels, for all ***x*** ∈ *X*_*L*_, where 1 ≤ *i* ≤ 3, indicating the Cartesian components). Let us denote this point by ***x***_***g***_, the target point of the global estimation phase.

As mentioned earlier, colonial walk cannot employ the full search space while learning the regression tree, because of the high computational cost. We must learn the target point from the whole CT volume because it is the first phase and no hints are available to determine the point of initialization. The point of initialization should be assigned randomly among any of the points inside the whole volume. Therefore, we use the whole range of CT volumes as training datasets after applying a uniform sampling to reduce the sample space and, the computational complexity. The training samples for this phase can thus be denoted by

$$\begin{array}{ccc} S_{g}=\{\left(v,x,\hat{u}\right)|0\leq x_{i}<max\left(x_{i}\in V_{v}\right), & & \\ x_{i}\;\mathrm{mod}\;sstep=0,1\leq i\leq 3\} & \end{array}$$ (11)

where *v* is the CT volume index, ***x*** = (*x*_1_, *x*_2_, *x*_3_) is the voxel offset, $\hat{u}$ is the unit direction of ***x*** to the target point, ***x***_***g***_ and *sstep* is the search step or sampling period.

We should use a relatively larger search step (i.e., sparse sampling) to reduce the search space and improve the computation time, which might not result in an accurate localization of the target point as argued earlier. However, we can allow certain flexibility in this phase of localization because the detected point only needs to be inside the surrounding area and eligible to be the point of initialization for the next phase. We allowed a maximum error of 30 voxels measured in Euclidean distance.

We also need to propose the feature offset parameter, ***θ*** = (***v***_**1**_,***v***_**2**_), carefully within a certain range because these are the feature descriptor and the key decision maker in our learning process. The appropriate voxel-pair that should describe the feature of a sample is defined by these parameters. A small-ranged parameter proposal means that we are interested in defining a sample by the voxels located close to it. The useful feature descriptors of a sample are usually the nearly located voxels. Therefore, defining a small range in proposing (***v***_**1**_,***v***_**2**_) improves the localization accuracy. However, a small-ranged proposal reduces feature variation, resulting in slow learning. It is preferable to use an extended range of feature parameters in this phase to accelerate the learning process. For learning in the global estimation phase in our experiment, we used ***θ*** = (***v***_**1**_,***v***_**2**_), where ***v***_**1**_ ***∈P***_***g***_,***v***_**2**_ ***∈P***_***g***_. Here, ***P***_***g***_ = {(*p*_*x*_, *p*_*y*_, *p*_*z*_)∣|*p*_*x*_| ≤ 80, |*p*_*y*_| ≤ 80, |*p*_*z*_| ≤ 40}. *p*_*x*_, *p*_*y*_, *p*_*z*_ are the components along X, Y and Z-axis of parameter ***p***. The range along the Z-axis is relatively small because the voxel-spacing along the Z-axis (i.e., the inter-slice spacing) is larger.

We learn a regression tree for global estimation phase, using the defined training samples and feature parameter range, which can provide us with a direction for an input sample to reach the target point, ***x***_***g***_. Therefore, the proposed colonial walk can exploit the trained tree to reach ***x***_***g***_. Experiments showed that our resultant point in this phase was always inside a cube of 25 × 25 × 25 voxels around ***x***_***g***_. Let us denote the resultant point by $x_{g}^{\prime }$. This point will serve as a point of initialization in the second phase of the proposed model.

#### Local estimation phase

This is our final phase, where we learn to detect all of the landmarks locally using the colonial walk. The detected point in the global phase, $x_{g}^{\prime }$, serves as the point of initialization for detecting all landmarks in this phase. We learn each of the landmarks independently from a significantly smaller area around the corresponding landmark. Hence, the target point in this phase is the individual landmark, and the search space is a small area around the corresponding landmark, which also needs to be sufficiently large to include $x_{g}^{\prime }$. Let us denote the target point as ***x***_***l***_, where ***x***_***l***_ ***∈X***_***L***_, the set of all the landmarks. A cuboid of 160 × 160 × 80 voxels around any ***x***_***l***_ should be large enough to accommodate $x_{g}^{\prime }$. Hence, the training samples for the local estimation phase are the voxels inside that cube centered at the considered landmark. Though 150 × 150 × 60 cube can include all the landmarks in it, a bigger cube is used to ensure safety on including the detected global point despite the detection error.

The primary goal of this phase is to accurately localize the landmarks. Therefore, we use small-ranged feature offset parameters to use the nearby voxels as the feature descriptor of a sample, to ensure better accuracy. Thus, the reduced feature parameter space exploited in our experiment can be denoted by ***P***_***l***_ = {(*p*_*x*_, *p*_*y*_, *p*_*z*_)∣|*p*_*x*_| <= 40, |*p*_*y*_| <= 40, |*p*_*z*_| <= 20}. We train eight different regression trees to learn the directions to eight landmarks. The colonial walk initiates multiple walkers around the detected point in the global phase, and reaches each landmark using the corresponding tree.

## Results

We received CCTA images from 71 different patients who were of 53 ± 16 years old, about 34% of the studies being female. The images were captured using different parameter settings and the corresponding ground truth positions for the eight landmarks were provided by the experts. The number of slices was 310 on average, each slice having 512 × 512 voxels. Among the CCTA artifacts, the coronary artery motion artifact was prominent in a number of volumes. Among the 71 volumes, 31 volumes are captured for preoperative planning of 31 TAVI undergoing patients, containing significant valvular calcification. Among the remaining 40 volumes, a mild calcification was observed in 11 studies. Calcification of aortic stenosis can affect the valvular area in unpredictable ways. To report the robustness of the proposed method, we conduct our evaluation in two experiments. In the first experiment, we performed a four-fold cross-validation on the 40 non-TAVI volumes and tested the trained models on the 31 volumes of TAVI patients. The second experiment follows the final cross-validation on the entire dataset of 71 volumes. For each test case, we applied the proposed colonial walk along with the random tree walk to localize the landmarks, using the same regression tree, so that we can compare the proposed method with the original RTW. We report the localization error for each landmark. We also show a comparison of the aortic annulus diameters and annulus to ostia distance, obtained from the resultant landmarks and the ground truth.

**Fig 7:**
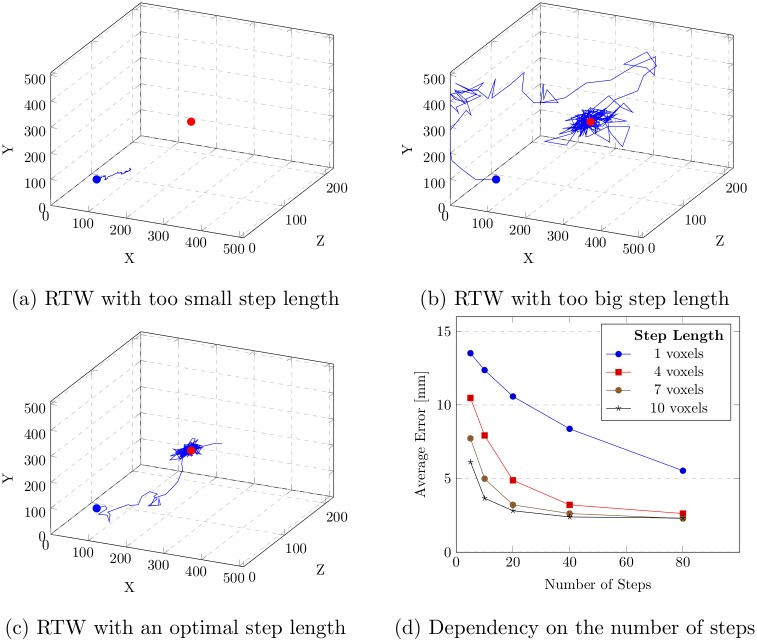
The dependency of the average localization error (in mm) on the step length (in voxels) and the number of steps. The blue and red dots in (a), (b) and (c) are the initial and the target ground truth positions, respectively. The walker fails to reach the target because of too small step length. Scattered movement is noticed for a very big step length. Relatively smooth movement is noticed for an optimal step length. (d) Dependency of the average localization error on the total number of steps for different step lengths.

To choose an optimal step size, we tested the localization performance of a single tree walk in local phase by assigning different step sizes. The non-coronary hinge point is used as the target landmark used for this experiment. The localization error is calculated in terms of the Euclidean distance (in mm) between the detected landmark and the target landmark. Fig 7 shows our observation, where the dependency on step size and total number of steps is shown. The step sizes are assigned in voxel units. The error decreases with the increase in step size and remains almost constant after a certain step size for a specific number of steps. It again increases after a certain step size. The rate of error fluctuation is reduced with the increase in the total number of steps.

**Fig 8:**
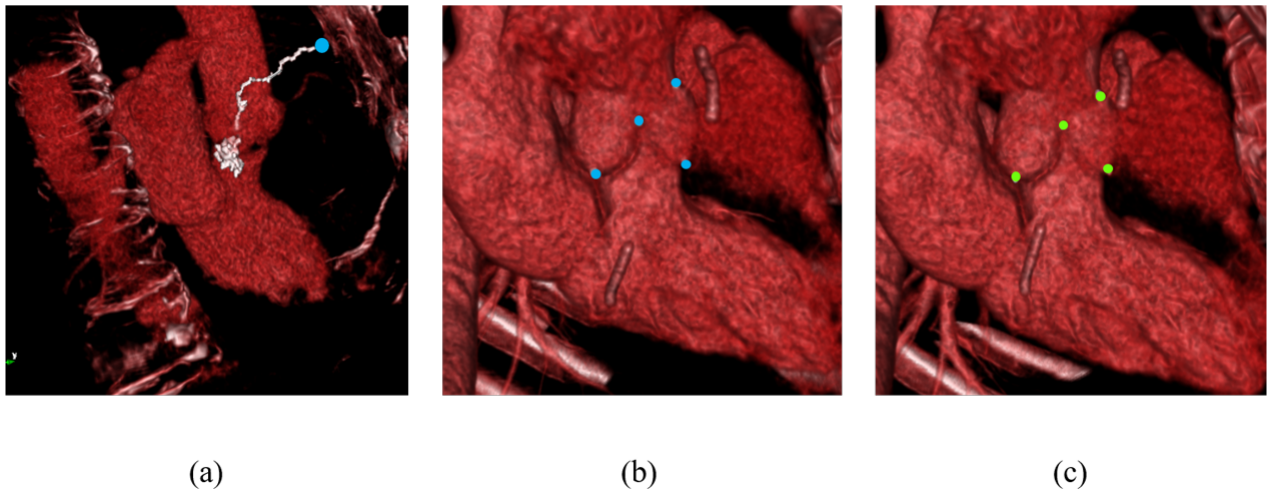
Colonial walk-localized landmarks in a test volume of a normal patient during the cross-validation on 40 non-TAVI volumes. (a) Successful walk with the minimum walk variance towards the non-coronary hinge point in global phase. The blue point refers to the initial position of the walker. (b) Local estimation of the non-coronary and right-coronary hinge points, the commissure point between them, and the right coronary ostium. (c) Corresponding ground truth.

**Fig 9:**
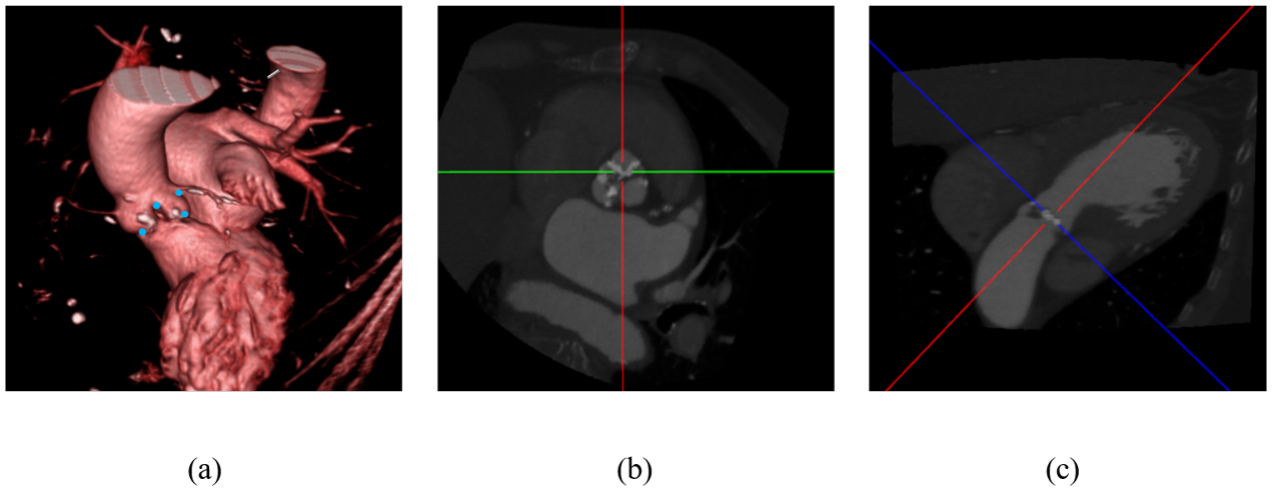
Localized landmarks in a TAVI volume using the models trained on 40 non-TAVI volumes. (a) Localized landmarks in volumetric view. (b) Short axis view. (c) LVOT view.

Nine regression trees were trained (one for the global estimation phase, eight for the local estimation phase) for each of the experiments. We applied both RTW and the proposed colonial walk for each test case. Each case is repeated multiple times to get an average performance for both method. During a test session, both RTW and the colonial walk first exploit the global phase tree to estimate the globally representative point. Using their own detected global point as the initial beginning, they exploit eight trees individually to estimate the eight landmarks position. Fig 8 shows the qualitative results of the proposed method in a test volume for the first experiment, where both our localized position and ground truth position exist. Fig 9 shows the localized landmarks in a TAVI volume using the trees trained on 40 non-TAVI volumes during the initial cross-validation. Multiple walkers taking different pathways from different side increased the probability of reaching the target in valvular feature variation due to calcification.

**Fig 10:**
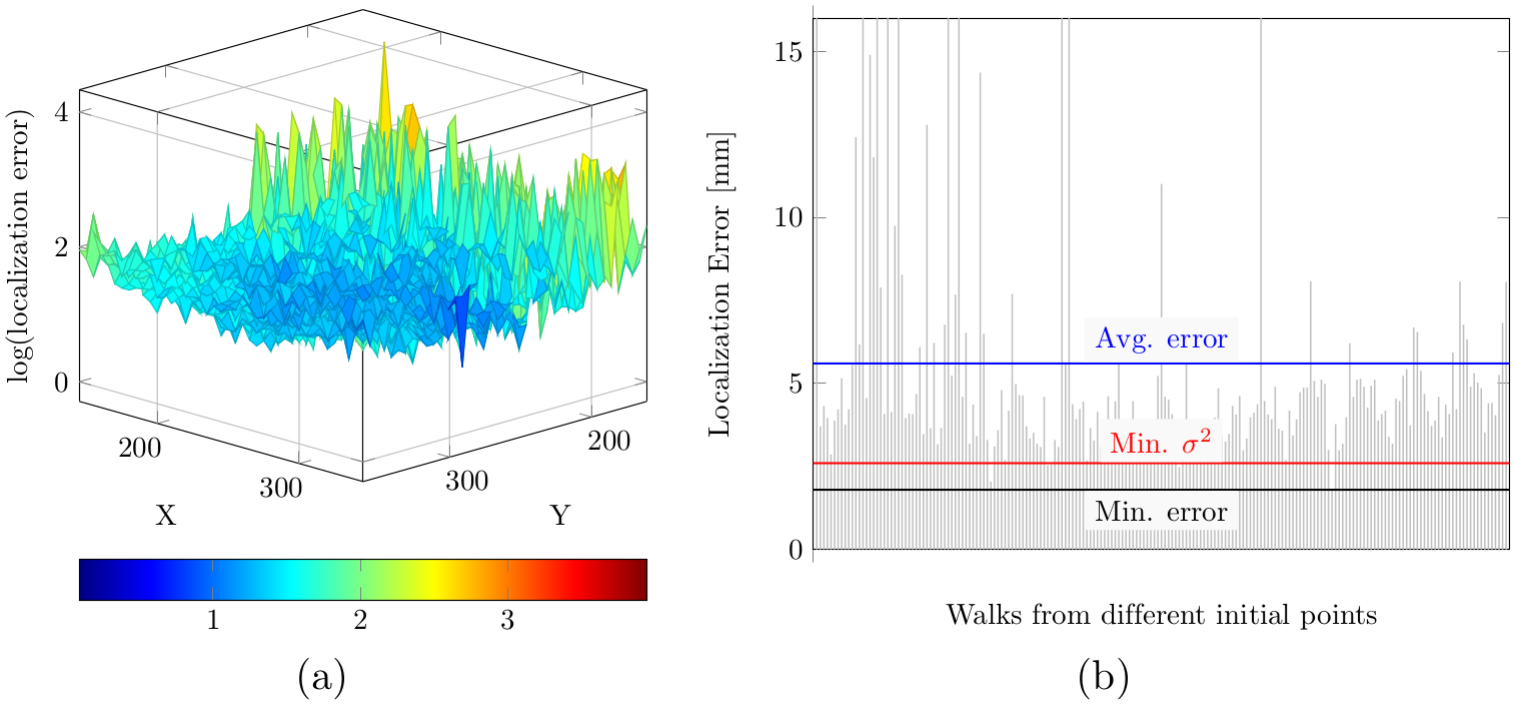
Localization error for different initial points in an unknown TAVI volume exploiting the knowledge of non-TAVI volumes only. (a) Logarithm of the localization error for different initial points on an axial slice near the target. (b) flattened and sampled representation of the corresponding localization error. The average error and minimum error is indicated in contrast with the error triggered by the minimum walk variance.

In order to evaluate our method quantitatively, we calculated the localization error as the euclidean distance (in mm) between the localized landmark and the ground truth. Table 2 presents the average localization error of the aortic hinges, commissures, and coronary ostia, for both the proposed method and RTW case in the first experiment on 40 non-TAVI volumes. Table 3 reports the results of applying the same trained models for localizing in the 31 TAVI volumes. The proposed method showed a remarkable outcome in coping with the variation resulting from the calcification, despite being trained only on the non-TAVI volumes, where a large error is noticed for the RTW. The utilization of walks from multiple initial points and the walk variance measure enables the colonial walk to explore more stable trajectories to the target. Fig 10 plots the localization error corresponding to the initial points sampled from an axial slice near the target landmark. Despite leading close to the target landmark, the average RTW performance is not satisfactory, whereas the walk variance could extract a near optimal walk. Table 4 presents the final cross-validation results on 71 volumes. The final mean localization error was 2.04 ± 1.11 mm for the proposed method. Here, the error is presented in *mean* ± *SD* (standard deviation) form. We could not directly compare our result with the existing methods because they used dataset obtained from different image modalities. However, the proposed method for CCTA has a noteworthy outcome, whereas [20]’s result in CTA was 2.65 ± 1.57 mm, where they did not localize the commissure points, and [17]’s result in C-arm CT was 2.11 ± 1.34 mm. The error differences between the proposed method and the RTW are statistically significant, showing a p-value less than 0.05.

**Table 2:** Fourfold cross validation test results on 40 non-TAVI volumes.

Localization error (mm)	Colonial walk	RTW
Hinge points	1.90 ± 0.84	2.05 ± 0.97
Commissure points	1.95 ± 0.93	2.12 ± 1.22
Coronary ostia	1.98 ± 1.07	2.18 ± 1.61
Overall error	1.94 ± 0.93	2.11 ± 1.22

**Table 3:** Localization results in 31 TAVI volumes using the models trained on 40 non-TAVI volumes.

Localization error (mm)	Colonial walk	RTW
Hinge points	2.81 ± 1.85	5.45 ± 3.30
Commissure points	2.77 ± 1.82	5.28 ± 3.16
Coronary ostia	2.54 ± 1.60	4.63 ± 2.82
Overall error	2.74 ± 1.78	5.19 ± 3.13

**Table 4:** Final cross-validation test results on 71 volumes.

Localization error (mm)		Colonial walk	RTW
Non-TAVI	Hinge points	2.01 ± 1.07	2.21 ± 1.26
	Commissure points	1.98 ± 1.02	2.16 ± 1.22
	Coronary ostia	2.01 ± 1.04	2.18 ± 1.25
	Overall error	1.99 ± 1.04	2.18 ± 1.24
TAVI	Hinge points	2.12 ± 1.28	2.68 ± 1.85
	Commissure points	2.10 ± 1.21	2.52 ± 1.84
	Coronary ostia	2.05 ± 1.08	2.44 ± 1.72
	Overall error	2.09 ± 1.20	2.56 ± 1.81
Inter-observer difference in CTA [20]		2.38 ± 1.56	

According to [20], the paired distance between two human observers marking the landmark in CTA was 2.38 ± 1.56 mm. It took about 4 minutes for the expert to customize the view and accurately marking all the landmarks in a CCTA volume. Our method has a high computational efficiency taking only 12 milliseconds on a 3.60 GHz single-core CPU, to localize all the landmarks, where no multi-thread parallelization is used. The population of the colony was 200. The average cosine distance between the normal vectors of the human-customized view-planes and of the view-planes obtained by the algorithm, was about 0.007 with a standard deviation of 0.004.

**Fig 11:**
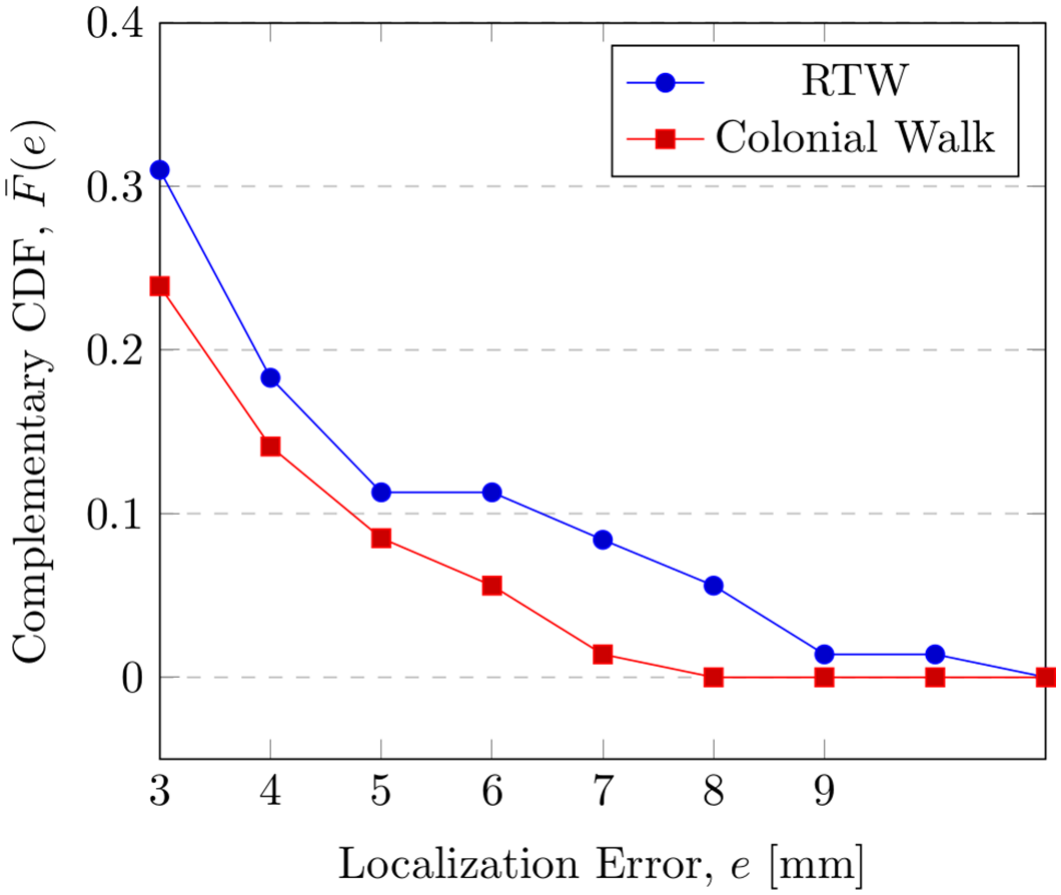
Complementary cumulative distribution function (CCDF) of the high localization error cases. $\bar{F}\left(e\right)=P\left[X\geq e\right]$ refers to the probability of error being greater than *e*. The colonial walk has reduced the probability of high error cases improving the localization for problematic volumes in RTW.

The proposed colonial walk method showed noteworthy improvement in localization performance over the RTW. It has reduced the average error and error variance for all of the hinges, commissures, and coronary ostia. Localization in calcified volumes was essentially improved. We observed a major improvement for the volumes that were exhibiting high localization error in case of RTW. As shown in Fig 11, our method was able to reduce the number of high error cases remarkably. The volume with high error could now find the most consistent walk with the help of the walk variance measure, ensuring a better localization.

The diameter of the aortic annulus is an important parameter for choosing the appropriate size of the prosthetic valve. Therefore, we also evaluated our method to obtain the aortic annulus diameter using the final experimental models. We calculate the annulus diameter as the diameter of the circle fitting the three hinges. We compare the diameters obtained from the localized hinge points and the ground truth position of the hinges. For getting an average evaluation, the circle fitting method is approached despite the annulus being irregularly elliptical. We also estimated the average distance of left and right coronary ostium from the annulus plane (i.e, the plane passing through three hinges). Table 5 presents the error of both parameters from the ground truth.

**Table 5:** Estimation error (in mm) of sizing parameters obtained from the localized landmarks.

Sizing parameter	Non-TAVI volumes	TAVI volumes
Aortic annulus diameter	0.88 ± 0.71	0.94 ± 0.83
Annulus to ostia distance	0.91 ± 0.76	0.98 ± 0.90

Annulus to ostia distance refers to the average distance of the left and right coronary ostium from the annulus plane.

**Fig 12:**
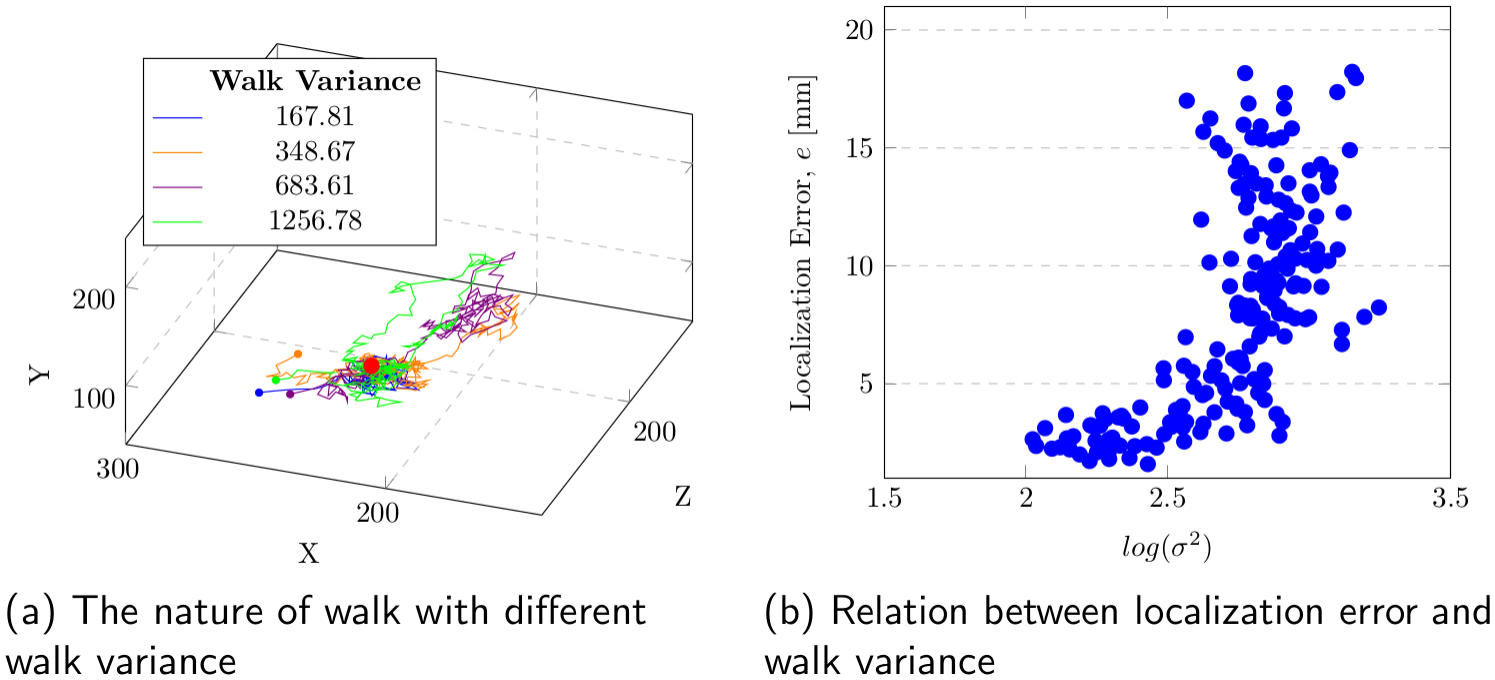
Walk variance relation to the localization error. (a) High error for high variance walk and low error for low variance walk is observed. (b) Localization error is presented against logarithm of walk variance.

The walk variance is the key factor in improving the localization performance in the proposed method, where small walk variance indicates a better guided walk. To observe the relationship between walk variance and localization error, we tested with 200 walks in Fig 12. Consistent walk was observed for a low walk variance. Relation of logarithmic walk variance to localization error is shown. Walk variance provides distinguishing information before a certain level. However, at a higher walk variance range, it does not provide any useful discrimination because error fluctuation is very high in that range.

**Fig 13:**
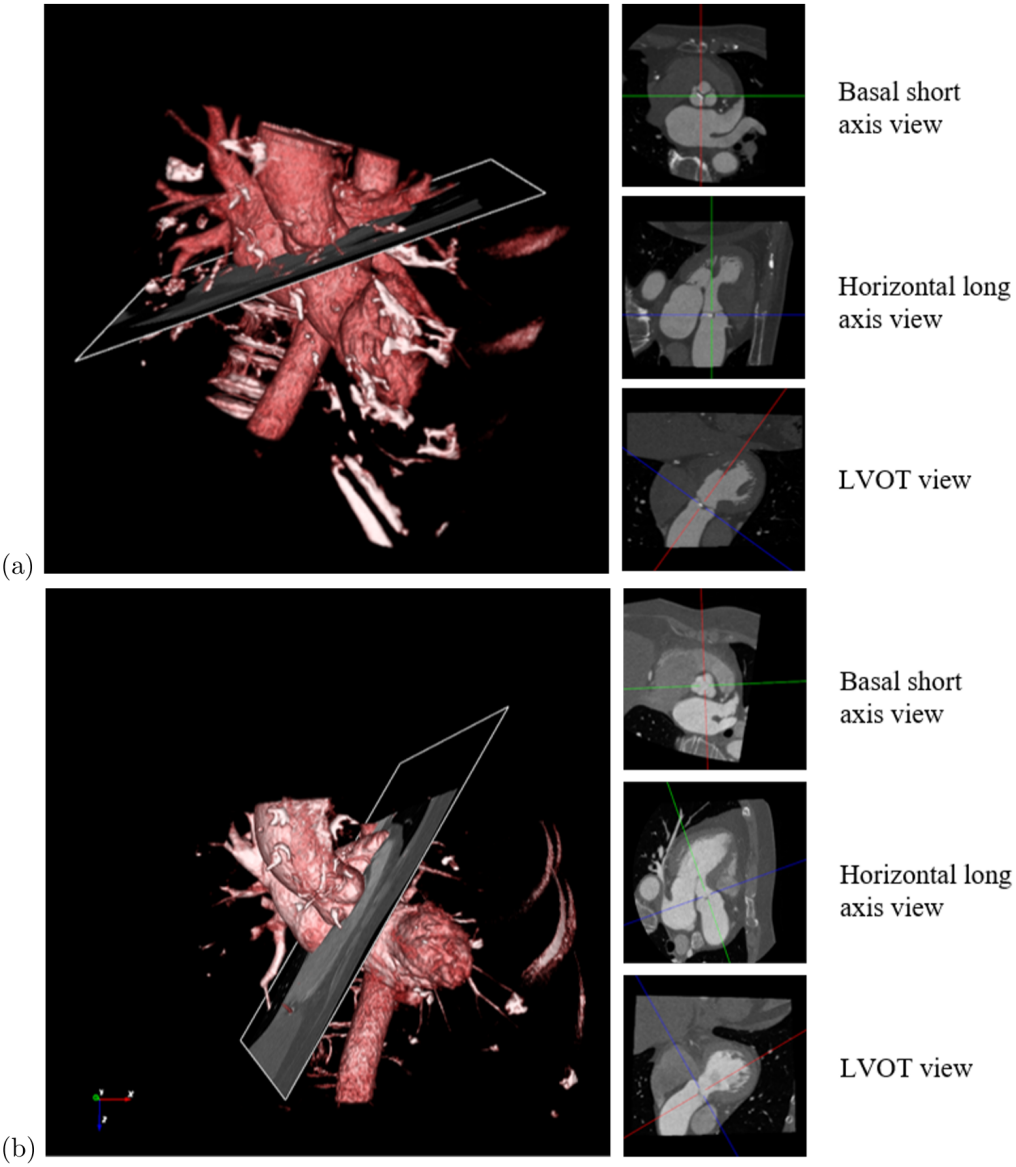
The case of high localization error. (a) A volume with typical error. (b) A volume with high error. Red, green and blue lines indicate the X, Y and Z axes, respectively. Rotation about X and Y axes is applied to have the axial view-plane parallel to the hinge plane for both volume to compare. The amount of rotation about Y-axis in case of the volume with high error was significantly higher comparing to the case of the volume with typical error.

While testing with RTW, we observed a high error for a set of certain volumes in localizing all of the landmarks. Fig 13 shows different views of such volume alongside the low-error-volume. In both cases, we rotated the axial plane about X and Y axes to axial view parallel to the hinge plane. The exceptional volumes could be described as a rotational transformation of the usual volumes. The rotational difference was significant about Y-axis. In those volumes, RTW had a greater chance of being misguided. However, in the case of the proposed method, multiple walkers are attempting to find their way into the target from multiple positions. Therefore, even in an exceptional volume, it can provide a better guided-walk, which can be chosen by the walk variance.

## Discussion

This paper presents an automatic method named colonial walk for localizing eight landmarks of the aortic valve in CT images, which can speed up the pre-procedural surgical planning of transcatheter aortic valve implantation (TAVI), and guide the physicians to have a quick customized view to analyze. A regression tree is trained to learn the direction at each voxel to the target landmark. In colonial walk, a colony of random walker starts from multiple initial points inside a test volume and takes steps towards the learned direction at that point. By taking such steps continuously, the random walker starts moving around the target. The expectation of the walker positions becomes the target landmark position. Thus each walker from the colony makes their own proposition of target position. We introduced the walk variance measure to choose the successful walker. The target position of the walker with the minimum walk variance becomes the resultant target position. A two-phase optimized search space model is proposed for efficient learning, where a representative point inside the valvular area is first learned, followed by learning all eight landmarks individually inside that area so that the colonial walk can first reach the representative point and then detect all other landmarks from that point. We observed a high accuracy in the fourfold cross validation on 71 CCTA volumes, 31 of them being acquired from TAVI undergoing patients. The proposed method has a high efficiency taking only 12 milliseconds to localize all the landmarks, where no multi-thread parallelization is used. The proposed method showed noteworthy improvement over the random tree walk (RTW), especially for the volumes showing high error in RTW.

## Supporting information

S1 File Uploaded code for the proposed colonial walk. (TXT)
